# Association between the serum sodium-to-chloride ratio and 30-day mortality following coronary artery bypass grafting: insights from the MIMIC-IV database

**DOI:** 10.1186/s12872-026-05830-9

**Published:** 2026-04-16

**Authors:** Zhisheng Yan, Yu Qiao, Haixia Sun, Huihui Cao, Wanteng Ma, Qing Chang

**Affiliations:** 1 Department of Cardiovascular Surgery, The Affiliated Hospital of Qingdao University, No. 16 Jiangsu Road, Qingdao, Shandong 266003 China; 2 Pediatric Clinic, Qingdao Municipal Hospital, Qingdao, Shandong China; 3 Healthcare Clinic, The Affiliated Hospital of Qingdao University, Qingdao, Shandong China

**Keywords:** Sodium-to-Chloride Ratio, 30-Day Mortality, Coronary Artery Bypass Grafting, MIMIC-IV

## Abstract

**Background:**

Abnormalities in electrolyte balance are common following coronary artery bypass grafting (CABG) and are associated with adverse outcomes. The serum sodium-to-chloride ratio has emerged as a potential prognostic marker in critical illness, but its role in predicting short-term mortality after CABG remains unclear. This study explored the relationship between the serum sodium-to-chloride ratio and 30-day mortality following CABG, aiming to assess the predictive value of the sodium-to-chloride ratio for short-term mortality and provide insights for optimizing postoperative electrolyte management.

**Methods:**

A retrospective cohort study was carried out using the Medical Information Mart for Intensive Care IV (MIMIC-IV) database, covering intensive care unit (ICU) admissions from 2008 to 2022 at the Beth Israel Deaconess Medical Center (BIDMC). The sodium-to-chloride ratio, measured within 24 h and divided into tertiles, was the exposure variable, while 30-day mortality was the outcome. Covariates included demographics, comorbid conditions, physiological scores, vital signs, and laboratory results. Multivariable Cox regression, restricted cubic splines (RCS), subgroup analyses, and doubly robust estimation (IPTW combined with Cox regression) were used to evaluate the independent association, dose–response relationship, and the robustness of the results.

**Results:**

Patients in the highest tertile of sodium-to-chloride ratio had a significantly higher risk of 30-day mortality than those in the lowest tertile. In the fully adjusted model, each 0.01-unit increase in the sodium-to-chloride ratio was associated with a 6% higher mortality risk (HR = 1.06, 95% CI: 1.02–1.11). Kaplan–Meier analysis showed lower survival rates in the highest tertile (*p* = 0.038). RCS demonstrated a significant association between the sodium-to-chloride ratio and 30-day mortality (P for overall = 0.011), with no evidence of non-linearity (P for non-linearity = 0.565), suggesting a linear relationship. Subgroup analysis showed a significant interaction in the MBP subgroup (p for interaction < 0.05), but not in other subgroups. Sensitivity analysis using doubly robust estimation yielded consistent results.

**Conclusions:**

An elevated serum sodium-to-chloride ratio within 24 h is independently associated with increased 30-day mortality after CABG, showing a linear dose–response relationship. These findings can guide clinicians in optimizing electrolyte management strategies to improve the short-term outcomes.

**Graphical Abstract:**

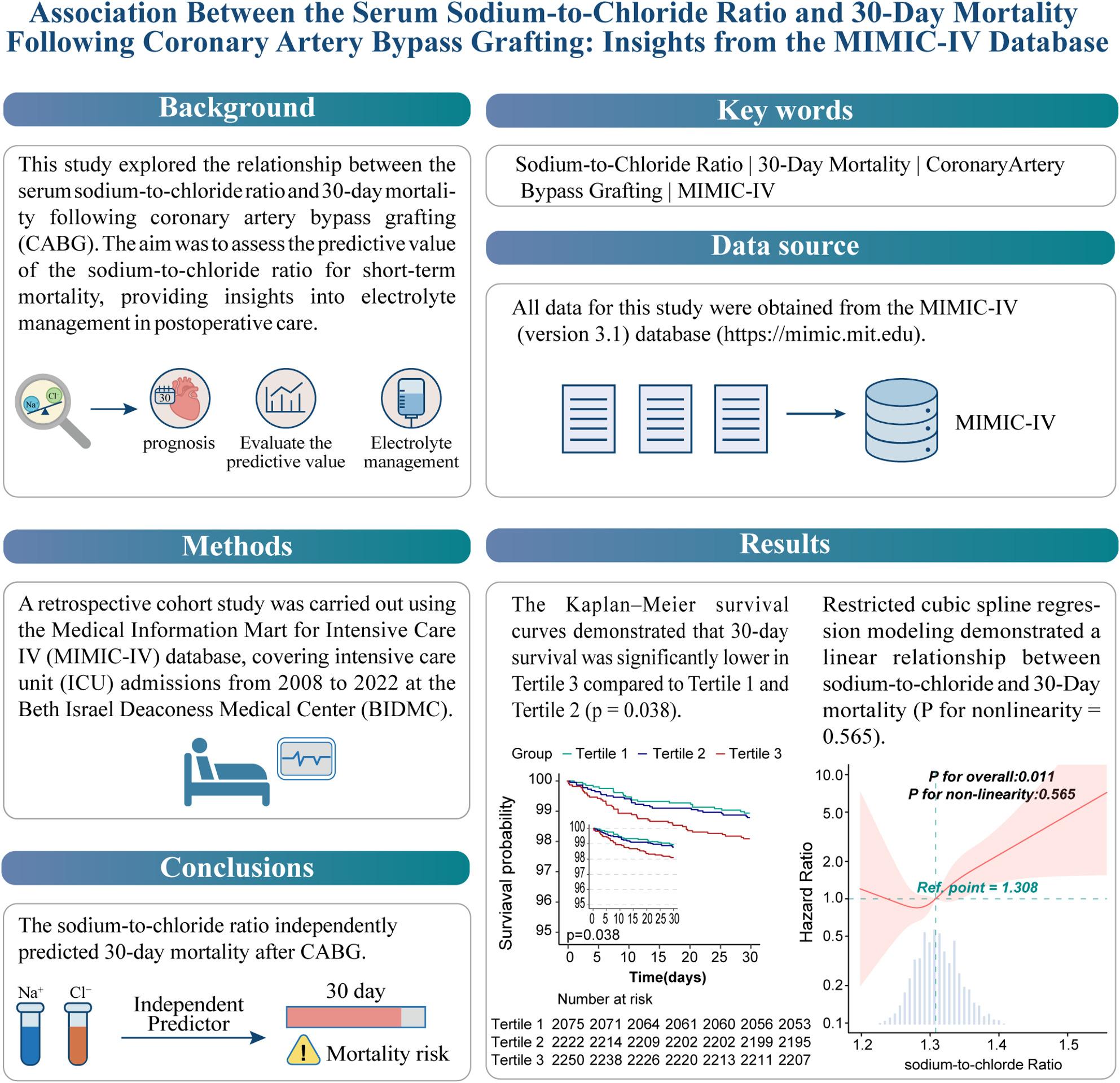

**Supplementary Information:**

The online version contains supplementary material available at 10.1186/s12872-026-05830-9.

## Introduction

Coronary artery bypass grafting (CABG) is a major surgical procedure for treating coronary heart disease. Based on data from the Society of Thoracic Surgery (STS) database [1], the mortality rate after CABG surgery, especially the 30-day mortality rate, remains approximately 2%. Despite improvements in surgical techniques and perioperative management, short-term mortality after CABG varies among countries. In China, especially in the context of aging and the high prevalence of cardiovascular diseases, the number of CABG surgeries has been increasing year by year, and the postoperative 30-day mortality rate is usually around 2.5%-4%, suggesting that the short-term prognosis of postoperative patients remains a concern.

The serum sodium-to-chloride ratio has emerged as a novel integrated electrolyte marker that captures disturbances in fluid status, strong ion difference, and systemic acid-base equilibrium. As such, it reflects a more comprehensive physiological state than isolated sodium or chloride measurements alone. Previous studies have demonstrated that abnormal elevation of this ratio is associated with poor prognosis in patients with acute heart failure [2]. The pathophysiological mechanism of an abnormal sodium-chloride ratio may be related to extracellular fluid regulation, electrolyte balance imbalance, and cardiac arrhythmia, but its specific role is still under study.

Existing studies indicate a correlation between abnormalities in serum sodium and chloride levels and adverse cardiovascular events and outcomes [3]. However, the relationship between the sodium-to-chloride ratio and short-term mortality following CABG has not yet been systematically explored. Therefore, investigating the predictive value of the sodium-to-chloride ratio for 30-day mortality after CABG is crucial for enhancing patient outcomes.

Accordingly, we designed this retrospective cohort study to investigate the association between the early postoperative sodium-to-chloride ratio and 30-day mortality following CABG. We hypothesized that an elevated ratio would be independently associated with increased short-term mortality. The present study aims to expand the current evidence base linking electrolyte balance to postoperative outcomes in CABG patients and to inform evidence-based strategies for perioperative electrolyte monitoring and management.

## Materials and methods

### Data source

All data for this study were obtained from the MIMIC-IV (version 3.1) database (https://mimic.mit.edu), a publicly accessible, real-world database containing extensive clinical information from over 65,000 ICU patients at BIDMC (a large tertiary care hospital in Boston, MA, USA), spanning from 2008 to 2022 [4]. The use of this database for research was approved by the Massachusetts Institute of Technology Affiliates and the BIDMC Institutional Review Board. Zhisheng Yan has completed the “Conflicts of Interest” (Record ID: 64546027) and “Data or Specimens Only Research” (Record ID: 64546026) exams, as well as the Collaborative Institutional Training Initiative (CITI) course, which provided access for data extraction. The Declaration of Helsinki’s tenets and the Strengthening the Reporting of Observational Studies in Epidemiology (STROBE) Statement’s recommendations were followed in this investigation.

### Study population

Participants in the study had to be admitted to the intensive care unit (ICU) and have had CABG, as defined by the Ninth and Tenth Revisions of the International Classification of Diseases (ICD-9 and ICD-10), according to the MIMIC-IV database. For patients with multiple hospital admissions due to CABG or multiple ICU admissions in the MIMIC-IV database, only the first admission was included in the analysis. The exclusion criteria were as follows: (1) missing sodium data, (2) missing chloride data, and (3) missing 30-day mortality data.

### Exposure variable

The first serum sodium and chloride levels were measured within 24 h after CABG. The sodium-to-chloride ratio, calculated by dividing serum sodium by serum chloride, was utilized to classify patients into three tertiles based on their admission levels. This classification aimed to decipher the association between the sodium-to-chloride ratio and 30-day mortality following CABG.

### Covariates

For this study, data collected within the first 24 h of ICU admission were used as the primary measurement parameters for analysis. A selection of covariates was retrieved from the MIMIC-IV database for analysis. The covariates included are as follows: (1) Demographics: age and gender; (2) Comorbidity conditions: myocardial infarction, congestive heart failure, cerebrovascular disease, chronic pulmonary disease, diabetes, and renal disease; (3) Scoring system: Charlson Comorbidity Index (CCI) and Acute Physiology Score III(APS III) ; (4) Vital signs: heart rate, mean blood pressure (MBP), Respiratory rate (RR), and saturation of peripheral oxygen (SPO_2_); (5) Laboratory examination: white blood cell (WBC), platelet count, hemoglobin, potassium, urea nitrogen, and glucose. All variables were extracted from the MIMIC-IV database using Structured Query Language (SQL) and Navicat software (version 17.0.13).

### Outcomes

The outcome was 30-day mortality following CABG.

### Statistical analysis

In this study, continuous variables were summarized as means ± standard deviation (SD) for normally distributed data, or as medians with interquartile ranges (IQR) for data with non-normal distribution. Categorical variables were presented as frequencies or percentages. Differences between groups were evaluated using the Pearson chi-square test or Fisher’s exact test for categorical variables. For continuous variables, the one-way analysis of variance (ANOVA) was applied to those with a normal distribution, while the Kruskal-Wallis test was used for continuous variables with skewed distributions. Missing data for covariates were imputed using the K-nearest neighbors method. Multivariable Cox proportional hazards regression models were used to assess the independent association between the sodium-to-chloride ratio and 30-day mortality. Three models were constructed: Model I, adjusted for age and gender; Model II, further adjusted for myocardial infarction, congestive heart failure, cerebrovascular disease, chronic pulmonary disease, diabetes, renal disease, CCI, and APSⅢ; and Model III, additionally adjusted for heart rate, MBP, RR, SpO₂, WBC, platelet count, hemoglobin, potassium, urea nitrogen, and glucose. Kaplan–Meier survival curves were used to visualize 30-day mortality across sodium-to-chloride ratio tertiles. Subgroup analyses were conducted based on factors including age, gender, MBP, myocardial infarction, congestive heart failure, cerebrovascular disease, chronic pulmonary disease, diabetes, and renal disease. To further explore the individual contributions of serum sodium and chloride, we separately categorized serum sodium and chloride into tertiles and examined their associations with 30-day mortality using the same multivariable adjustment strategy. Non-linearity was tested using restricted cubic splines (RCS) with a likelihood ratio test comparing the model with only a linear term against the model including linear and spline terms. All statistical tests were two-sided, with a p-value of less than 0.05 considered statistically significant. Hazard ratios (HR) and 95% confidence intervals (CI) were reported. Statistical analyses were performed using R 4.4.2 (https://www.r-project.org, The R Foundation) and Free Statistics software (version 2.0).

## Results

### Population selection and baseline characteristics

**Fig. 1:**
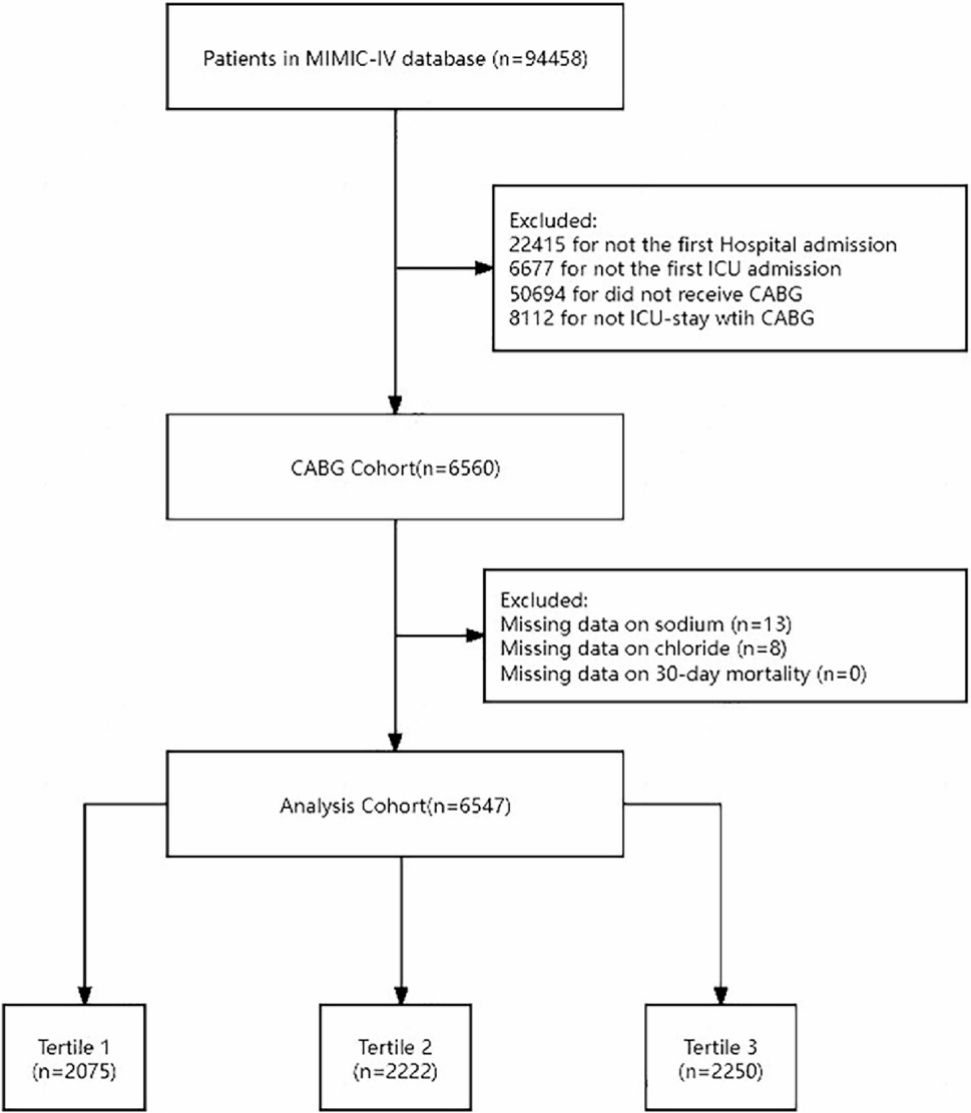
Flowchart of population selection

In this study, 6547 patients who received CABG were included according to the various exclusion criteria. Patiens selection process is illustrated in Fig. 1. Table 1 shows the baseline population characteristics categorized into three groups according to sodium-to-chloride ratio tertiles: Tertile 1 (1.137 ~ 1.292), Tertile 2 (1.292 ~ 1.324), and Tertile 3 (1.324 ~ 1.557). The median age of the population was 68.6 ± 10.2 years. Of the patients, 78.4% were men (*n* = 5,134) and 21.6% were women (*n* = 1,413). Among the three groups, there were no significant statistical differences in chronic pulmonary disease, diabetes, renal disease, and heart rate (*P* > 0.05). However, significant statistical differences were observed in age, gender, myocardial infarction, congestive heart failure, cerebrovascular disease, CCI, APSⅢ, MBP, RR, SPO_2_, WBC, platelet count, hemoglobin, potassium, urea nitrogen, glucose, and 30-day mortality. Notably, the 30-day mortality was significantly higher in Tertile 3 (1.9%, *p* = 0.038) compared to Tertile 1 (1.1%) and Tertile 2 (1.2%).

**Table 1:** Baseline characteristics of participants according to sodium-chloride ratio

Variables	Total (*n* = 6547)	Sodium-Chloride Ratio			*p*
		T1 (*n* = 2075)	T2 (*n* = 2222)	T3 (*n* = 2250)	
Demographics					
Age (years)	68.6 ± 10.2	70.2 ± 10.1	68.5 ± 10.0	67.3 ± 10.3	< 0.001
Gender, n (%)					< 0.001
Female	1413 (21.6)	510 (24.6)	449 (20.2)	454 (20.2)	
Male	5134 (78.4)	1565 (75.4)	1773 (79.8)	1796 (79.8)	
Comorbidity conditions					
Myocardial infarction, n (%)	2682 (41.0)	838 (40.4)	838 (37.7)	1006 (44.7)	< 0.001
Congestive heart failure, n (%)	1607 (24.5)	511 (24.6)	506 (22.8)	590 (26.2)	0.027
Cerebrovascular disease, n (%)	683 (10.4)	267 (12.9)	212 (9.5)	204 (9.1)	< 0.001
Chronic pulmonary disease, n (%)	1192 (18.2)	393 (18.9)	392 (17.6)	407 (18.1)	0.536
Diabetes, n (%)	2751 (42.0)	865 (41.7)	918 (41.3)	968 (43)	0.478
Renal disease, n (%)	1187 (18.1)	391 (18.8)	389 (17.5)	407 (18.1)	0.523
Scoring system					
CCI	5.4 ± 2.2	5.6 ± 2.1	5.3 ± 2.1	5.3 ± 2.2	< 0.001
APSⅢ	34.0 (27.0, 45.0)	35.0 (28.0, 46.5)	34.0 (26.0, 43.0)	33.0 (26.0, 45.8)	< 0.001
Vital signs					
Heart rate, (bpm)	81.7 ± 9.6	81.9 ± 9.0	81.7 ± 9.3	81.6 ± 10.3	0.504
MBP, (mmHg)	74.8 ± 6.6	74.3 ± 6.4	74.5 ± 6.4	75.6 ± 7.0	< 0.001
RR, (bpm)	17.8 ± 2.6	17.5 ± 2.4	17.8 ± 2.4	18.1 ± 2.7	< 0.001
SPO_2_, (%)	97.7 ± 1.5	97.9 ± 1.3	97.7 ± 1.3	97.4 ± 1.7	< 0.001
Laboratory examination					
WBC, (×10^9^/L)	12.8 ± 5.8	12.5 ± 4.1	12.9 ± 7.5	12.9 ± 5.2	0.037
Platelet Count, (×10^9^/L)	163.2 ± 56.3	158.3 ± 56.1	161.5 ± 52.4	169.6 ± 59.4	< 0.001
Hemoglobin, (g/dL)	10.3 ± 1.5	10.2 ± 1.4	10.3 ± 1.4	10.5 ± 1.6	< 0.001
Potassium, (mmol/L)	4.4 ± 0.5	4.5 ± 0.5	4.4 ± 0.4	4.3 ± 0.5	< 0.001
Urea Nitrogen, (mg/dL)	16.0 (12.0, 20.0)	15.0 (12.0, 20.0)	15.0 (12.0, 20.0)	16.0 (12.0, 21.0)	0.03
Glucose, (mg/dL)	129.8 (121.9, 140.5)	129.0 (121.2, 139.1)	130.1 (122.6, 140.1)	130.2 (121.8, 141.8)	0.002
Outcomes					
30-day mortality, n (%)	92 (1.4)	22 (1.1)	27 (1.2)	43 (1.9)	0.038

*CCI* Charlson comorbidity index, *APSⅢ *Acute physiology score III, *MBP *Mean blood pressure, *RR* Respiratory rate, *SPO*_2_ Saturation of peripheral oxygen, *WBC* White blood cell, *AKI* Acute kidney injury

### Multivariable Cox regression analysis and sensitivity analysis

In this study, three models were constructed to analyze the independent association between the sodium-to-chloride ratio and 30-day mortality. We found that the effect sizes remained robust across the crude and adjusted models (*p* < 0.05). In the crude model, each 0.01-unit increase in the sodium-to-chloride ratio was associated with an 11% higher risk of 30-day mortality (HR = 1.11, 95% CI: 1.06–1.16). In the adjusted models, each 0.01-unit increase in the sodium-to-chloride ratio corresponded to a 12% increase in 30-day mortality in Model I (HR = 1.12, 95% CI: 1.07–1.17), a 7% increase in Model II (HR = 1.07, 95% CI: 1.03–1.12), and a 6% increase in Model III (HR = 1.06, 95% CI: 1.02–1.11). To enhance the sensitivity analysis, we converted the continuous sodium-to-chloride ratio into a categorical variable, dividing it into tertiles. Tertile 1 served as the baseline reference. Patients in the highest tertile (Tertile 3) of the sodium-to-chloride ratio showed an increased 30-day mortality risk compared to those in Tertile 1 and Tertile 2. Notably, the trend p-value for the fully adjusted model, with sodium-to-chloride ratio categorized into tertiles, aligned with the trend observed when this variable was treated as continuous (*p* < 0.05) (Table 2). Additionally, we performed a sensitivity analysis that excluded missing data for covariates to ensure the robustness of the results (Supplementary Table S1). To further evaluate the potential confounding effect of lactate, we conducted another sensitivity analysis by including lactate level as an adjusted covariate in the multivariable Cox regression model (Supplementary Table S2). Finally, the results of the doubly robust estimation were consistent with the main analysis (Supplementary Table S3), indicating that our findings were robust. The Kaplan–Meier survival curves after excluding covariates with missing values were consistent with the primary results (Supplementary Figure S1). Overall, these sensitivity analyses supported the robustness and reliability of our findings.

**Table 2:** Multivariable cox regression to assess the association between sodium-to-chloride ratio and 30-day mortality

Exposure	Crude Model	*P*	Model Ⅰ	*P*	Model Ⅱ	*P*	Model Ⅲ	*P*
	HR (95%CI)		HR (95%CI)		HR (95%CI)		HR(95%CI)	
sodium-to-chloride ratio^a^	1.11 (1.06 ~ 1.16)	< 0.001	1.12 (1.07 ~ 1.17)	< 0.001	1.07 (1.03 ~ 1.12)	0.001	1.06 (1.02 ~ 1.11)	0.006
sodium-to-chloride ratio tertiles								
Tertile 1	Reference		Reference		Reference		Reference	
Tertile 2	1.15 (0.65 ~ 2.02)	0.632	1.29 (0.74 ~ 2.27)	0.373	1.44 (0.82 ~ 2.53)	0.208	1.3 (0.74 ~ 2.3)	0.364
Tertile 3	1.81 (1.08 ~ 3.03)	0.023	2.13 (1.27 ~ 3.56)	0.004	1.99 (1.18 ~ 3.34)	0.009	1.78 (1.04 ~ 3.05)	0.036
*P* for trend		0.017		0.003		0.009		0.033

Sodium-to-chloride ratio^a^ was entered as continuous variable per 0.01 increase; Crude Model: didn’t adjusted for covariates; Model Ⅰ: adjusted for age, gender; Model Ⅱ: Model Ⅰ+myocardial infarction, congestive heart failure, cerebrovascular disease, chronic pulmonary disease, diabetes, renal disease, CCI, and APSⅢ; Model Ⅲ: Model Ⅱ+heart rate, MBP, RR, SPO_2_, WBC, platelet count, hemoglobin, potassium, urea nitrogen, and glucose

### Exploratory analyses of individual sodium and chloride

To further evaluate the individual effects of serum sodium and chloride, we performed exploratory analyses using multivariable Cox model, examining both continuous and tertile-based categorizations, along with RCS analysis. All models were adjusted for the same covariates as in the primary analysis.

Serum sodium was significantly associated with 30-day mortality in the multivariable Cox model (Supplementary Table S4). RCS analysis further confirmed this association (P for overall = 0.001) and revealed a non-linear relationship (P for non-linearity = 0.007), as shown in Supplementary Figure S2. serum chloride was not associated with 30-day mortality in the multivariable Cox model (Supplementary Table S5). Consistently, restricted cubic spline analysis showed no significant association (P for overall = 0.125), as shown in Supplementary Figure S3. In contrast, the sodium-to-chloride ratio showed a more consistent and robust association across multiple modeling approaches. Detailed results are presented in Table 2; Fig. 4.

### Subgroup analysis

**Fig. 2:**
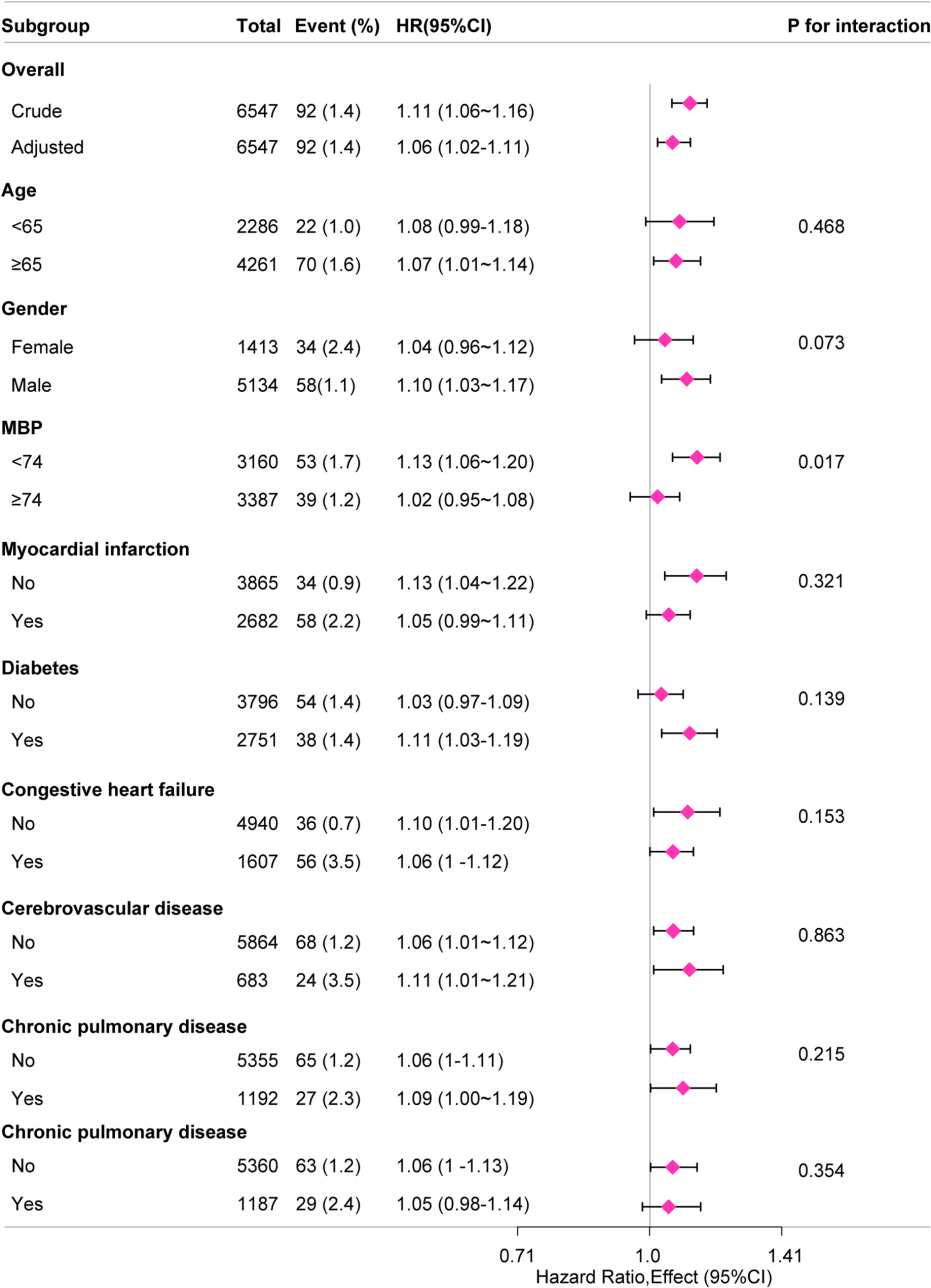
The relationship between sodium-to-chloride and 30-day mortality in subgroup analysis

To assess the impact of the sodium-to-chloride ratio on 30-day mortality in different subgroups, a subgroup analysis was conducted. The results revealed a significant interaction in the MBP subgroup (p for interaction < 0.05), while no significant interactions were found in the other subgroups (p for interaction > 0.05). Moreover, a positive correlation was found. (Table 3; Fig. 2)

**Table 3:** Subgroup analysis of the association between sodium-to-chloride ratio and 30-day mortality

Subgroup	Total	Event (%)	HR (95%CI)	*P* for interaction
Overall				
Crude	6547	92 (1.4)	1.11 (1.06 ~ 1.16)	
Adjusted	6547	92 (1.4)	1.06 (1.02 ~ 1.11)	
Age				
<65	2286	22 (1.0)	1.08 (0.99 ~ 1.18)	0.468
≥65	4261	70 (1.6)	1.07 (1.01 ~ 1.14)	
Gender				
Female	1413	34 (2.4)	1.04 (0.96 ~ 1.12)	0.073
Male	5134	58 (1.1)	1.10 (1.03 ~ 1.17)	
MBP				
<74	3160	53 (1.7)	1.13 (1.06 ~ 1.20)	0.017
≥74	3387	39 (1.2)	1.02 (0.95 ~ 1.08)	
Myocardial infarction				
No	3865	34 (0.9)	1.13 (1.04 ~ 1.22)	0.321
Yes	2682	58 (2.2)	1.05 (0.99 ~ 1.11)	
Diabetes				
No	3796	54 (1.4)	1.03 (0.97 ~ 1.09)	0.139
Yes	2751	38 (1.4)	1.11 (1.03 ~ 1.19)	
Congestive heart failure				
No	4940	36 (0.7)	1.10 (1.01 ~ 1.20)	0.153
Yes	1607	56 (3.5)	1.06 (1.00 ~ 1.12)	
Cerebrovascular disease				
No	5864	68 (1.2)	1.06 (1.01 ~ 1.12)	0.863
Yes	683	24 (3.5)	1.11 (1.01 ~ 1.21)	
Chronic pulmonary disease				
No	5355	65 (1.2)	1.06 (1.00 ~ 1.11)	0.215
Yes	1192	27 (2.3)	1.09 (1.00 ~ 1.19)	
Renal disease				
No	5360	63 (1.2)	1.06 (1.00 ~ 1.13)	0.354
Yes	1187	29 (2.4)	1.05 (0.98 ~ 1.14)	

### Kaplan–Meier survival curves

**Fig. 3:**
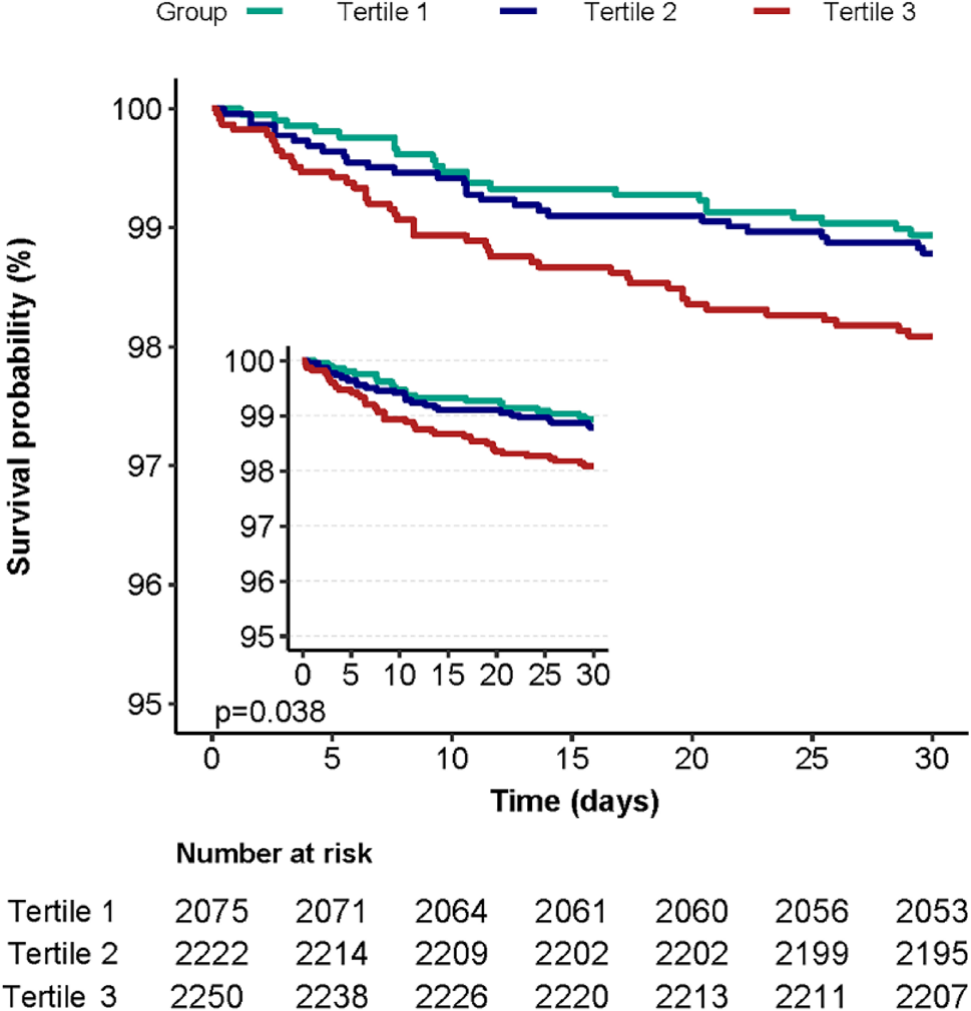
Kaplan-Meier survival curves

The Kaplan–Meier survival curves demonstrated that 30-day survival was significantly lower in Tertile 3 compared to Tertile 1 and Tertile 2 (*p* = 0.038, Fig. 3).

### Restricted cubic spline analysis

**Fig. 4:**
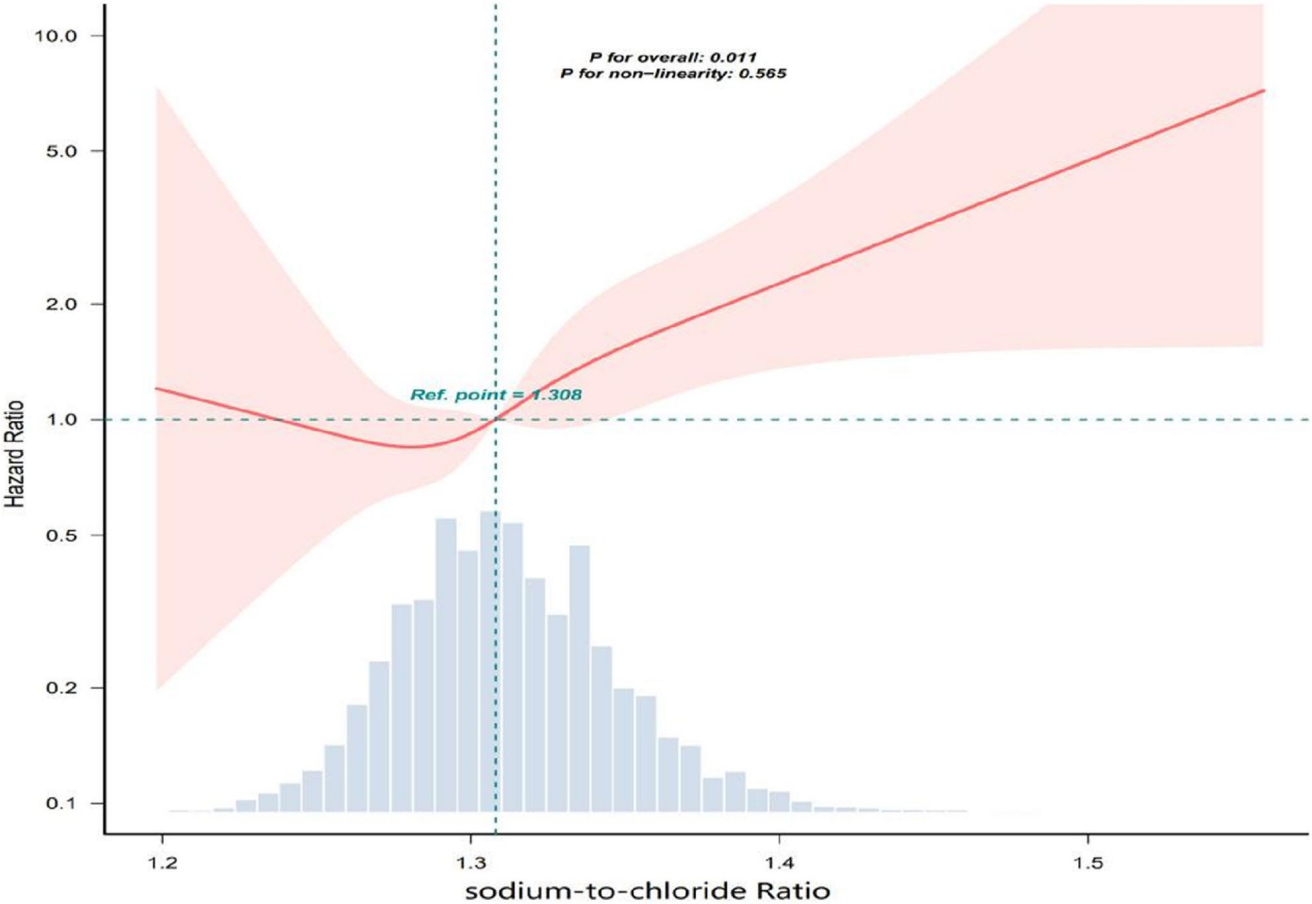
Restricted cubic spline analysis

Restricted cubic spline analysis demonstrated a significant association between the sodium-to-chloride ratio and 30-day mortality (P for overall = 0.011), with no evidence of non-linearity (P for non-linearity = 0.565), suggesting a linear relationship, as shown in Fig. 4.

## Discussion

Coronary artery disease (CAD) is basically characterized by the formation of atherosclerotic plaques within the walls of coronary arteries, resulting in narrowing of the vessel lumen, thrombosis, and even vessel occlusion, leading to angina attacks, myocardial infarction, and heart failure, which has led to cardiovascular disease becoming the leading cause of death worldwide [5]. The main treatment modalities for CAD include pharmacological and invasive treatments, and CABG is an effective modality for blood flow reconstruction through repair or replacement of the occluded coronary arteries [6, 7].

CABG patients often have poor cardiac function after surgery, especially in CABG assisted by extracorporeal circulation, and there is a postoperative water-electrolyte-acid-base imbalance, which seriously affects patients’ prognosis. Electrolytes play a crucial role in maintaining cardiac function and hemodynamic stability, and electrolyte imbalances have been shown to play an important role in fluid homeostasis and in influencing the progression of CHF [8]. Electrolyte imbalances often involve serum sodium and chloride, which are important cations and anions in the extracellular fluid. These imbalances can interfere with cardiac electrical activity, affect fluid balance, and exacerbate heart failure [9]. It has been documented that even mild hyponatremia predicts adverse events, including decreased survival, rehospitalization, and prolonged hospital stays [10]. Hyponatremia [11] and hypochlorhydria [12] have each been linked to an increased risk of mortality, while research has also identified hypochlorhydria and hyperchloremia as independent risk factors for major adverse cardiovascular events (MACEs) [13–15].

However, the associations between individual electrolyte levels and clinical outcomes have not been entirely consistent across studies, particularly after adjustment for confounding factors and across different clinical settings. Variations in patient populations, perioperative management, and analytical approaches may partly explain these discrepancies [16, 17]. In line with this variability, when evaluated individually in our study, serum sodium exhibited a significant non-linear association with 30-day mortality, whereas serum chloride alone was not significantly associated with outcomes after multivariable adjustment. The lack of an independent association for serum chloride may be attributable to its sensitivity to perioperative fluid administration, dilutional effects, and acid–base disturbances, which could attenuate its prognostic value after adjustment. For example, chloride levels are strongly influenced by the administration of chloride-rich intravenous fluids (e.g., normal saline), which can induce hyperchloremic metabolic acidosis and alter renal perfusion, thereby confounding its direct relationship with outcomes [18–20]. Additionally, according to the Stewart strong ion theory, chloride plays a central role in acid–base balance, and fluctuations in chloride concentration often reflect compensatory physiological responses rather than primary pathological processes, potentially attenuating its independent prognostic value after adjustment [21, 22]. In contrast, serum sodium is more tightly regulated and reflects broader disturbances in water balance, neurohormonal activation, and hemodynamic status, which may explain its stronger and non-linear association with mortality [23]. These findings suggest that single electrolyte measurements may provide limited or unstable prognostic information when considered in isolation, particularly in the context of complex postoperative physiology.

The associations between serum sodium and chloride levels and clinical outcomes may vary after adjustment for covariates, including lactate, and further studies are warranted to better elucidate their relationships with patient prognosis. Lactate is a well-established biomarker of tissue hypoperfusion and has been consistently linked to increased mortality in critically ill and post–cardiac surgery populations [24, 25]. In the context of coronary artery bypass grafting (CABG), hyperlactatemia may result from perioperative hemodynamic instability, cardiopulmonary bypass–induced inflammatory responses, and impaired systemic oxygen delivery [25, 26]. Previous studies have focused solely on serum sodium and chloride, but changes in serum sodium are often accompanied by changes in serum chloride, for example, lower serum chloride levels usually coincide with lower serum sodium levels [27]. It has been shown that serum chloride is strongly associated with survival in patients with chronic heart failure and is a major contributor to the risk of hyponatremia [28]. Taken together, there is an interaction between the two, making it more meaningful to study the sodium-to-chlorine ratio. It has been shown that the sodium-to-chloride ratio is positively associated with in-hospital mortality in acute heart failure [29]. In the present study, we found that the serum sodium-to-chloride ratio is significantly associated with 30-day mortality following coronary artery bypass grafting in Tertile 3 (1.324 ~ 1.557). A higher sodium-to-chloride ratio was associated with an increased risk of 30-day mortality, which provides a new direction for postoperative management of CABG.

The mechanism by which a high sodium-to-chloride ratio increases mortality is not clear, but several explanations may account for this phenomenon: (1) Serum sodium and chloride are important cations and anions in electrolytes, and their imbalances can cause disorders in the internal environment, such as hyperchloremic acidosis, and decreased contractility of the heart; (2) Abnormalities in serum sodium can easily lead to cardiac arrhythmias, resulting in decreased cardiac function (3) Abnormalities in serum sodium and chloride increase the effective circulating blood volume, leading to hypertension, increased afterload, increased cardiac work, and decreased cardiac function. The mechanism may involve a family of serine-threonine kinases known as WNK [With-No-Lysine (K)], particularly WNK1 and WNK4. These kinases are recognized as crucial regulators of blood pressure and electrolyte balance. Mutations in WNK1 and WNK4 have been associated with hypertension, hyperchloremic metabolic acidosis, and hyperkalemia due to increased renal sodium chloride reabsorption [30–33]. In our study, a significant interaction was observed in the subgroup analysis of MBP (p for interaction < 0.05). The relationship between the sodium-to-chloride ratio and 30-day mortality was more pronounced in patients with lower MBP (< 74), with a hazard ratio (HR) of 1.13 (95% CI: 1.06–1.20). This finding highlights the need for closer clinical attention to this subset of patients.

Previous studies have not explored the relationship between the sodium-to-chloride ratio and short-term adverse outcomes following CABG. In our current research, we combined serum sodium and chloride measurements to comprehensively evaluate the association between the sodium-to-chloride ratio and 30-day mortality after CABG. This approach may provide better guidance for postoperative electrolyte management, including the use of diuretics and fluid volume control, potentially reducing the incidence of short-term adverse outcomes. We hope our findings will offer valuable insights for postoperative care and prognosis assessment.

Nevertheless, our study has certain limitations. As a retrospective cohort study, the results may be influenced by covariates and sample selection bias, limiting the ability to establish causality and underscoring the need for further in-depth research. Additionally, although our study included a large sample size, the findings are specific to CABG patients admitted to the ICU and may have limited generalizability to low-risk CABG patients managed outside the ICU. To determine the generalizability of our results, multicenter studies are needed to explore the impact of the sodium-to-chloride ratio on 30-day mortality following CABG.

## Conclusion

In conclusion, this study demonstrates a direct association between the sodium-to-chloride ratio and 30-day mortality following CABG. These findings underscore the critical interaction between serum sodium and chloride levels and provide a theoretical basis for addressing electrolyte imbalances after CABG.

## Supplementary Information

Supplementary Material 1: Table S1: Multivariate Cox Regression After Excluding Covariates with Missing Values. Figure S1: Kaplan-Meier Survival Curves After Excluding Covariates with Missing Values.

## Data Availability

All data was retrieved from MIMIC-IV (version 3.1) database (https://mimic.mit.edu). Access to the files requires being a credentialed user, completing the necessary training, and signing the data use agreement for the project. All data supporting this study can be obtained from the corresponding author upon request.
