# Supplementary material for: Association between the serum sodium-to-chloride ratio and 30-day mortality following coronary artery bypass grafting: insights from the MIMIC-IV database
Source: BMC Cardiovasc Disord. 2026 Apr 16;26:453. doi: 10.1186/s12872-026-05830-9 (PMC13214111; doi:10.1186/s12872-026-05830-9)
Supplement: Supplementary file 1 — Supplementary Material 1: Table S1: Multivariate Cox Regression After Excluding Covariates with Missing Values. Figure S1: Kaplan-Meier Survival Curves After Excluding Covariates with Missing Values. [file 12872_2026_5830_MOESM1_ESM.docx]

**Supplementary Materials**

**1. Table S1 Multivariate Cox Regression After Excluding Covariates with Missing Values**

| Exposure | Crude Model | *P* | ModelⅠ | *P* | ModelⅡ | *P* | ModelⅢ | *P* |
| --- | --- | --- | --- | --- | --- | --- | --- | --- |
|  | HR (95%CI) |  | HR (95%CI) |  | HR (95%CI) |  | HR(95%CI) |  |
| sodium-to-chloride ratio^a^ | 1.11 (1.06~1.16) | <0.001 | 1.12 (1.07~1.17) | <0.001 | 1.07 (1.03~1.12) | 0.001 | 1.06 (1.02~1.11) | 0.006 |
| sodium-to-chloride ratio tertiles |  |  |  |  |  |  |  |  |
| Tertile 1 | Reference |  | Reference |  | Reference |  | Reference |  |
| Tertile 2 | 1.1 (0.62~1.95) | 0.736 | 1.24 (0.7~2.19) | 0.46 | 1.38 (0.78~2.45) | 0.266 | 1.25 (0.7~2.22) | 0.45 |
| Tertile 3 | 1.8 (1.08~3.01) | 0.025 | 2.11 (1.26~3.53) | 0.005 | 1.98 (1.18~3.33) | 0.01 | 1.78 (1.04~3.04) | 0.037 |
| *P* for trend |  | 0.017 |  | 0.003 |  | 0.009 |  | 0.032 |

Note: Sodium-to-chloride ratio^a^ was entered as continuous variable per 0.01 increase; Crude Model: didn’t adjusted for covariates; ModelⅠ: adjusted for age, gender; ModelⅡ: ModelⅠ+ myocardial infarction, congestive heart failure, cerebrovascular disease, chronic pulmonary disease, diabetes, renal disease, CCI, and APSⅢ; Model Ⅲ: ModelⅡ+heart rate, MBP, RR, SPO2, WBC, platelet count, hemoglobin, potassium, urea nitrogen, and glucose.

**2. Table S2. Multivariable cox regression to assess the association between the sodium-to-chloride ratio and 30-Day mortality (Including lactate)**

| Exposure | Crude Model | *P* | Model Ⅰ | *P* | Model Ⅱ | *P* | Model Ⅲ | *P* |
| --- | --- | --- | --- | --- | --- | --- | --- | --- |
|  | HR (95%CI) |  | HR (95%CI) |  | HR (95%CI) |  | HR(95%CI) |  |
| sodium-to-chloride ratio^a^ | 1.11 (1.06~1.16) | <0.001 | 1.12 (1.07~1.17) | <0.001 | 1.07 (1.03~1.12) | 0.001 | 1.07 (1.02~1.12) | 0.003 |
| sodium-to-chloride ratio tertiles |  |  |  |  |  |  |  |  |
| Tertile 1 | Reference |  | Reference |  | Reference |  | Reference |  |
| Tertile 2 | 1.15 (0.65~2.02) | 0.632 | 1.29 (0.74~2.27) | 0.373 | 1.44 (0.82~2.53) | 0.208 | 1.12 (0.63~2.01) | 0.696 |
| Tertile 3 | 1.81 (1.08~3.03) | 0.023 | 2.13 (1.27~3.56) | 0.004 | 1.99 (1.18~3.34) | 0.009 | 1.77 (1.03~3.05) | 0.038 |
| *P* for trend |  | 0.017 |  | 0.003 |  | 0.009 |  | 0.031 |

Note: sodium-to-chloride ratio^a^ was entered as continuous variable per 0.01 increase; Crude Model: didn’t adjusted for covariates; Model Ⅰ: adjusted for age, gender; Model Ⅱ: Model Ⅰ+myocardial infarction, congestive heart failure, cerebrovascular disease, chronic pulmonary disease, diabetes, renal disease, CCI, and APSⅢ; Model Ⅲ: Model Ⅱ+heart rate, MBP, RR, SPO_2_, WBC, platelet count, hemoglobin, potassium, urea nitrogen, and glucose, lactate.

**3. Table S3. Association between sodium-to-chloride ratio tertiles and 30-day mortality across three analytical models**

| **Exposure** | **Crude Model** | ***P*** | **Multivariable HR** | ***P*** | **Doubly Robust HR** | ***P*** |
| --- | --- | --- | --- | --- | --- | --- |
|  | **HR (95%CI)** |  | **HR (95%CI)** |  | **HR**  **(95%CI)** |  |
| sodium-to-chloride ratio tertiles |  |  |  |  |  |  |
| Tertile 1 | Reference |  | Reference |  | Reference |  |
| Tertile 2 | 1.15 (0.65~2.02) | 0.632 | 1.3 (0.74~2.3) | 0.364 | 1.25 (0.97~1.62) | 0.472 |
| Tertile 3 | 1.81 (1.08~3.03) | 0.023 | 1.78 (1.04~3.05) | 0.036 | 1.79 (1.41~2.27) | 0.044 |
| *P* for trend |  | 0.017 |  | 0.003 |  | 0.039 |

**Note: Crude: unadjusted; Multivariable: adjusted for all covariates; Doubly Robust: doubly robust estimation using inverse probability of treatment weighting (IPTW) and multivariable Cox regression.**

**4. Figure S1 Kaplan-Meier Survival Curves After Excluding** **Covariates with Missing Values**


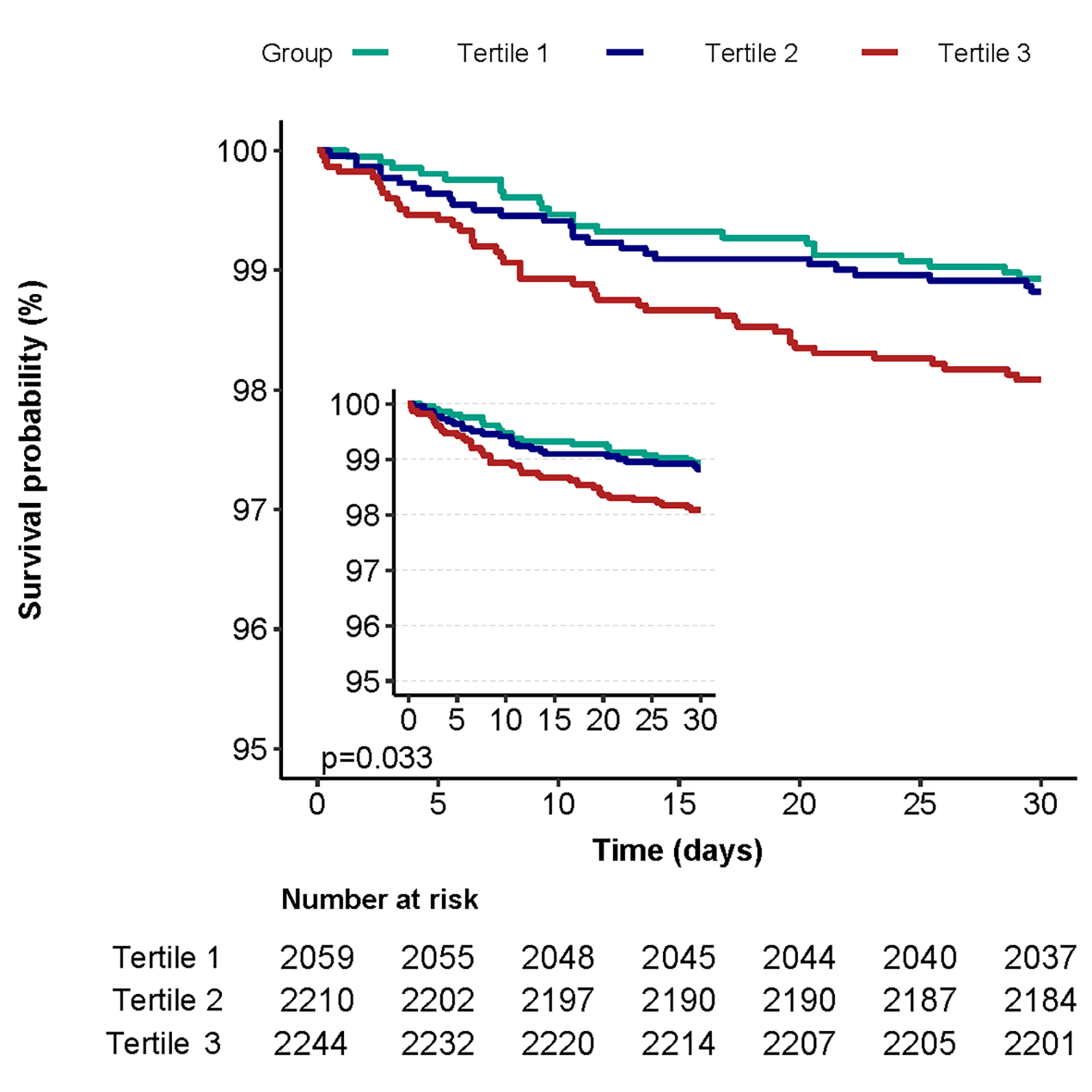


**5. Table S4. Multivariable cox regression to assess the association between serum sodium and 30-Day mortality**

| Exposure | Crude Model | *P* | Model Ⅰ | *P* | Model Ⅱ | *P* | Model Ⅲ | *P* |
| --- | --- | --- | --- | --- | --- | --- | --- | --- |
|  | HR (95%CI) |  | HR (95%CI) |  | HR (95%CI) |  | HR(95%CI) |  |
| sodium | 1.16 (1.08~1.24) | <0.001 | 1.14 (1.07~1.22) | <0.001 | 1.10 (1.04~1.17) | 0.001 | 1.09 (1.02~1.15) | 0.008 |
| sodium tertiles |  |  |  |  |  |  |  |  |
| Tertile 1 | Reference |  | Reference |  | Reference |  | Reference |  |
| Tertile 2 | 1.06  (0.57~2.00) | 0.845 | 1.10 (0.59~2.07) | 0.76 | 1.33 (0.71~2.50) | 0.375 | 1.41 (0.74~2.66) | 0.296 |
| Tertile 3 | 1.82 (1.08~3.08) | 0.025 | 1.79 (1.06~3.03) | 0.029 | 1.84 (1.09~3.11) | 0.023 | 1.75 (1.03~3.00) | 0.04 |
| *P* for trend |  | 0.013 |  | 0.017 |  | 0.019 |  | 0.038 |

Note: Crude Model: didn’t adjusted for covariates; Model Ⅰ: adjusted for age, gender; Model Ⅱ: Model Ⅰ+myocardial infarction, congestive heart failure, cerebrovascular disease, chronic pulmonary disease, diabetes, renal disease, CCI, and APSⅢ; Model Ⅲ: Model Ⅱ+heart rate, MBP, RR, SPO_2_, WBC, platelet count, hemoglobin, potassium, urea nitrogen, and glucose.

**6. Figure S2. Restricted cubic spline analysis for serum sodium**

Restricted cubic spline (RCS) analysis demonstrated a significant association between serum sodium and 30-day mortality (P for overall = 0.001), with evidence of a non-linear relationship (P for non-linearity = 0.007).


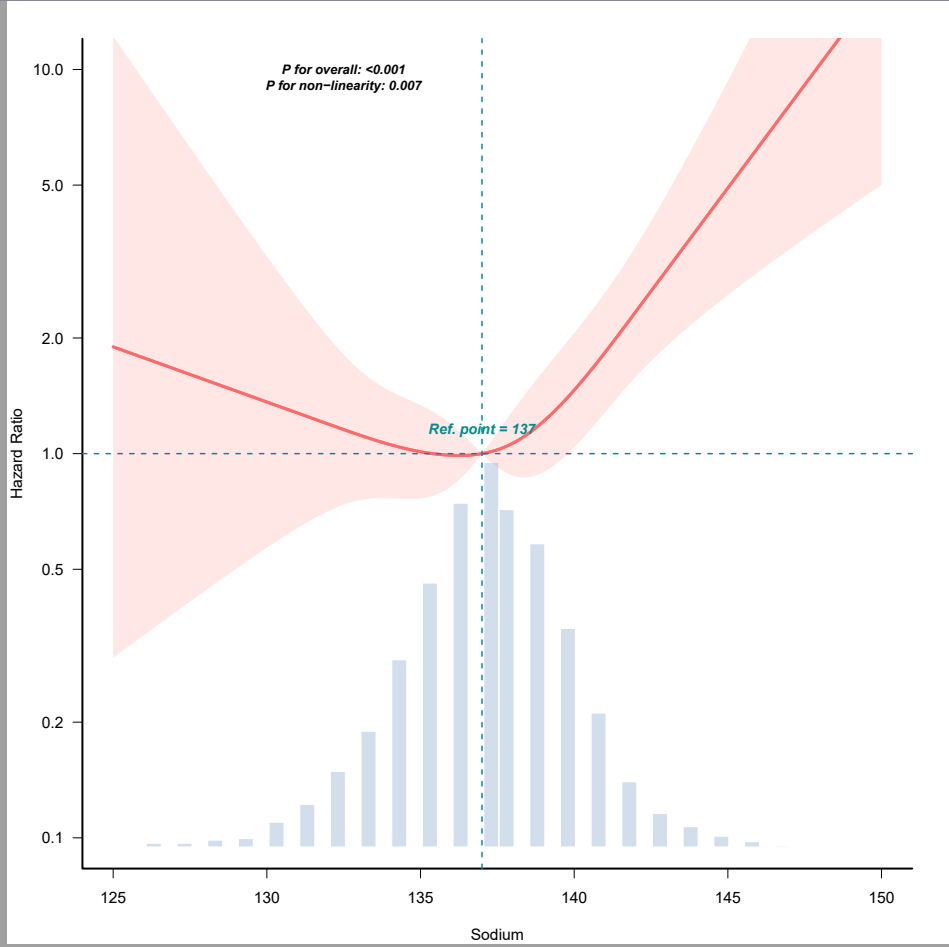


**7. Table S5. Multivariable cox regression to assess the association between serum chloride and 30-Day mortality**

| Exposure | Crude Model | *P* | Model Ⅰ | *P* | Model Ⅱ | *P* | Model Ⅲ | *P* |
| --- | --- | --- | --- | --- | --- | --- | --- | --- |
|  | HR (95%CI) |  | HR (95%CI) |  | HR (95%CI) |  | HR(95%CI) |  |
| chloride | 0.98 (0.93~1.03) | 0.484 | 0.97 (0.92~1.02) | 0.212 | 0.98 (0.94~1.03) | 0.522 | 0.99 (0.94~1.04) | 0.662 |
| chloride tertiles |  |  |  |  |  |  |  |  |
| Tertile 1 | Reference |  | Reference |  | Reference |  | Reference |  |
| Tertile 2 | 0.68  (0.40~1.17) | 0.166 | 0.67 (0.39~1.16) | 0.151 | 0.85 (0.49~1.48) | 0.569 | 0.85 (0.49~1.49) | 0.576 |
| Tertile 3 | 0.84 (0.52~1.34) | 0.458 | 0.74 (0.46~1.19) | 0.216 | 0.91 (0.56~1.47) | 0.699 | 0.93 (0.57~1.53) | 0.781 |
| *P* for trend |  | 0.532 |  | 0.259 |  | 0.728 |  | 0.817 |

Note: Crude Model: didn’t adjusted for covariates; Model Ⅰ: adjusted for age, gender; Model Ⅱ: Model Ⅰ+myocardial infarction, congestive heart failure, cerebrovascular disease, chronic pulmonary disease, diabetes, renal disease, CCI, and APSⅢ; Model Ⅲ: Model Ⅱ+heart rate, MBP, RR, SPO_2_, WBC, platelet count, hemoglobin, potassium, urea nitrogen, and glucose.

**8. Figure S3. Restricted cubic spline analysis for serum chloride**

Restricted cubic spline (RCS) analysis showed no significant association between serum chloride and 30-day mortality (P for overall = 0.125).


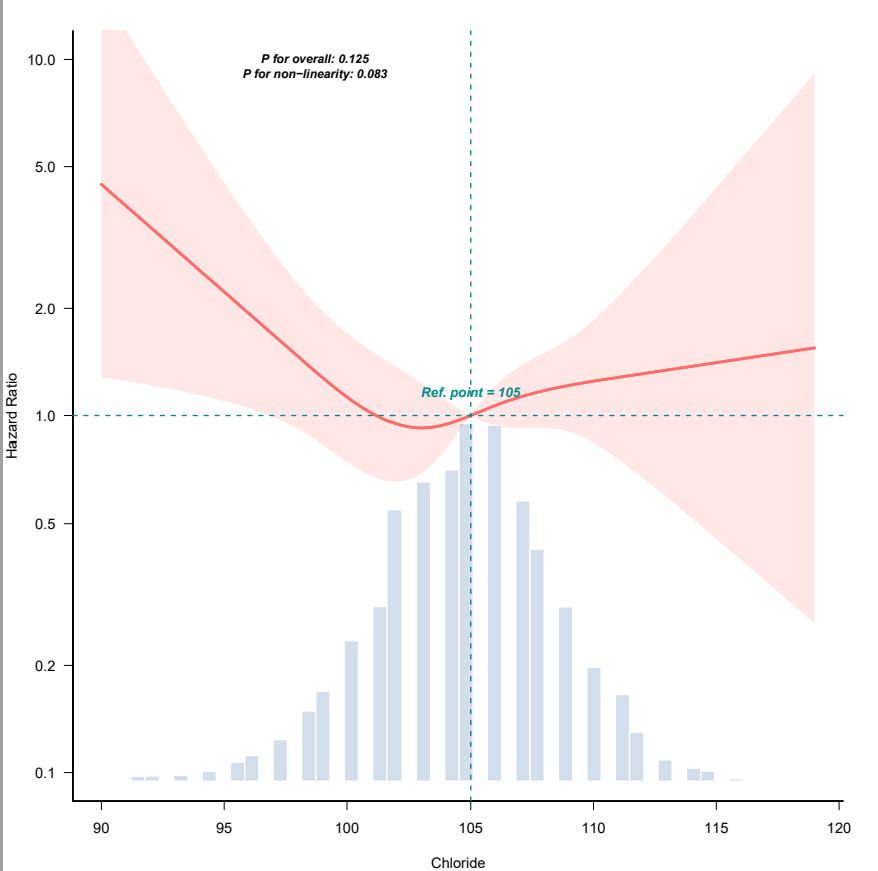


**9. Supplementary Table S6. The meaning of ICD-9 and ICD-10 codes for chronic pulmonary disease and renal disease**

| Variables | ICD | ICD codes | Meaning of diagnostic code |
| --- | --- | --- | --- |
| **chronic pulmonary disease** | ICD-9 | 490 | bronchitis, not specified as acute or chronic |
|  |  | 49121,49122,49120,4919,4918,4911 | chronic bronchitis |
|  |  | 4928,4920 | emphysema |
|  |  | 49390,49391,49320,49392,49322,49300,49381,49302,49382,49321,49301,49312,49310 | asthma |
|  |  | 4940,4941 | allergic alveolitis and pneumonitis |
|  |  | 4959,4958 | allergic alveolitis and pneumonitis |
|  |  | 496 | chronic airway obstruction, not elsewhere classified |
|  |  | 500 | coal workers' pneumoconiosis |
|  |  | 501 | asbestosis |
|  |  | 502 | pneumoconiosis due to other silica or silicates |
|  |  | 5060 | Bronchitis and pneumonitis due to fumes and vapors |
|  |  | 5081 | Chronic and other pulmonary manifestations due to radiation |
|  |  | 5088 | Respiratory conditions due to other specified external agents |
|  | ICD-10 | J40 | Bronchitis, not specified as acute or chronic |
|  |  | J410,J411 | chronic bronchitis |
|  |  | J42 | Unspecified chronic bronchitis |
|  |  | J430,J431,J432,J438,J439 | emphysema |
|  |  | J44,J440,J441,J449 | Chronic obstructive pulmonary disease |
|  |  | J45909,J45901,J45990,J45998,J45991,J45902 | asthma |
|  |  | J470,J471,J479 | bronchiectasis |
|  |  | J60 | coalworker's pneumoconiosis |
|  |  | J61 | pneumoconiosis due to asbestos and other mineral fibers |
|  |  | J62 | pneumoconiosis due to other dust containing silica |
|  |  | J632,J634,J636 | pneumoconiosis |
|  |  | J64 | unspecified pneumoconiosis |
|  |  | J668 | airway disease due to other specific organic dusts |
|  |  | J678,J679 | hypersensitivity pneumonitis |
|  |  | J684 | Chronic respiratory conditions due to chemicals, gases, fumes and vapors |
|  |  | J701 | Chronic and other pulmonary manifestations due to radiation |
|  |  | J703 | Chronic drug-induced interstitial lung disorders |

| Variables | ICD | ICD codes | Meaning of diagnostic code |
| --- | --- | --- | --- |
| **Renal disease** | ICD-9 | 5821,58281,5829,58289,5822 | Chronic glomerulonephritis |
|  |  | 5859,5851,5852,5853,5854,5855,5856 | Chronic kidney disease |
|  |  | 586 | Renal failure, unspecified |
|  |  | V560,V561,V562,V568,V5631 | other dialysis |
|  |  | 5880 | Renal osteodystrophy |
|  |  | V420 | Kidney replaced by transplant |
|  |  | V4511,V4512 | Renal dialysis status |
|  |  | 5830 | Nephritis and nephropathy, not specified as acute or chronic, with lesion of proliferative glomerulonephritis |
|  |  | 5831 | Nephritis and nephropathy, not specified as acute or chronic, with lesion of membranous glomerulonephritis |
|  |  | 5832 | Nephritis and nephropathy, not specified as acute or chronic, with lesion of membranoproliferative glomerulonephritis |
|  |  | 5834 | Nephritis and nephropathy, not specified as acute or chronic, with lesion of rapidly progressive glomerulonephritis |
|  |  | 5836 | Nephritis and nephropathy, not specified as acute or chronic, with lesion of renal cortical necrosis |
|  |  | 58381,5839,58389 | Nephritis and nephropathy |
|  |  | 40391,40310,40311,40301,40300 | Hypertensive chronic kidney disease |
|  |  | 40403 | Hypertensive heart and chronic kidney disease, malignant, with heart failure and with chronic kidney disease stage V or end stage renal disease |
|  |  | 40413 | Hypertensive heart and chronic kidney disease, benign, with heart failure and chronic kidney disease stage V or end stage renal disease |
|  |  | 40493,40492,40490 | Hypertensive heart and chronic kidney disease |
|  | ICD-10 | N189,N183,N186,N182,N184,N185,N1830,N1831,N1832,N181 | Chronic kidney disease |
|  |  | N19 | Unspecified kidney failure |
|  |  | I120 | Hypertensive chronic kidney disease with stage 5 chronic kidney disease or end stage renal disease |
|  |  | I1310,I1311 | Hypertensive heart and chronic kidney disease |
|  |  | N250 | Renal osteodystrophy |
|  |  | Z4901,Z4902 | Dialysis |
|  |  | Z940 | Kidney transplant status |
|  |  | Z992 | Dependence on renal dialysis |
|  |  | N032 | Chronic nephritic syndrome with diffuse membranous glomerulonephritis |
|  |  | N039 | Chronic nephritic syndrome with unspecified morphologic changes |
|  |  | N038 | Chronic nephritic syndrome with other morphologic changes |
|  |  | N050 | Unspecified nephritic syndrome with minor glomerular abnormality |
|  |  | N051 | Unspecified nephritic syndrome with focal and segmental glomerular lesions |
|  |  | N052 | Unspecified nephritic syndrome with diffuse membranous glomerulonephritis |
|  |  | N055 | Unspecified nephritic syndrome with diffuse mesangiocapillary glomerulonephritis |
|  |  | N057 | Unspecified nephritic syndrome with diffuse crescentic glomerulonephritis |
|  |  | N058 | Unspecified nephritic syndrome with other morphologic changes |
|  |  | N059 | Unspecified nephritic syndrome with unspecified morphologic changes |

Abbreviation: ICD: international classification of diseases.

**10. Supplementary Table S7. Multivariable logistic regression to assess the association between the sodium-to-chloride ratio and 30-Day mortality**

| Exposure | Crude Model | *P* | Model Ⅰ | *P* | Model Ⅱ | *P* | Model Ⅲ | *P* |
| --- | --- | --- | --- | --- | --- | --- | --- | --- |
|  | OR (95%CI) |  | OR (95%CI) |  | OR (95%CI) |  | OR  (95%CI) |  |
| sodium-to-chloride ratio^a^ | 1.11 (1.06~1.16) | <0.001 | 1.12 (1.07~1.18) | <0.001 | 1.08 (1.03~1.13) | 0.001 | 1.07 (1.02~1.13) | 0.005 |
| sodium-to-chloride ratio tertiles |  |  |  |  |  |  |  |  |
| Tertile 1 | Reference |  | Reference |  | Reference |  | Reference |  |
| Tertile 2 | 1.15 (0.65~2.02) | 0.633 | 1.3 (0.73~2.29) | 0.372 | 1.43 (0.8~2.57) | 0.229 | 1.33 (0.74~2.4) | 0.345 |
| Tertile 3 | 1.82 (1.08~3.05) | 0.023 | 2.14 (1.27~3.6) | 0.004 | 2.02 (1.18~3.45) | 0.011 | 1.81(1.04~3.15) | 0.037 |
| *P* for trend |  | 0.017 |  | 0.003 |  | 0.01 |  | 0.034 |

Note: sodium-to-chloride ratio^a^ was entered as continuous variable per 0.01 increase; Crude Model: didn’t adjusted for covariates; Model Ⅰ: adjusted for age, gender; Model Ⅱ: Model Ⅰ+myocardial infarction, congestive heart failure, cerebrovascular disease, chronic pulmonary disease, diabetes, renal disease, CCI, and APSⅢ; Model Ⅲ: Model Ⅱ+heart rate, MBP, RR, SPO_2_, WBC, platelet count, hemoglobin, potassium, urea nitrogen, and glucose.

**11. Supplementary Table S8. Multivariable cox regression to assess the association between the sodium-to-chloride ratio and 30-Day mortality, with additional adjustment for surgical type (on-pump vs off-pump).**

| Exposure | Crude Model | *P* | Model Ⅰ | *P* | Model Ⅱ | *P* | Model Ⅲ | *P* |
| --- | --- | --- | --- | --- | --- | --- | --- | --- |
|  | HR (95%CI) |  | HR (95%CI) |  | HR (95%CI) |  | HR(95%CI) |  |
| sodium-to-chloride ratio^a^ | 1.11 (1.06~1.16) | <0.001 | 1.12 (1.07~1.17) | <0.001 | 1.07 (1.03~1.12) | 0.001 | 1.08 (1.03~1.13) | 0.002 |
| sodium-to-chloride ratio tertiles |  |  |  |  |  |  |  |  |
| Tertile 1 | Reference |  | Reference |  | Reference |  | Reference |  |
| Tertile 2 | 1.15 (0.65~2.02) | 0.632 | 1.29 (0.74~2.27) | 0.373 | 1.44 (0.82~2.53) | 0.208 | 1.35 (0.76~2.39) | 0.301 |
| Tertile 3 | 1.81 (1.08~3.03) | 0.023 | 2.13 (1.27~3.56) | 0.004 | 1.99 (1.18~3.34) | 0.009 | 2.01 (1.15~3.49) | 0.014 |
| *P* for trend |  | 0.017 |  | 0.003 |  | 0.009 |  | 0.012 |

Note: sodium-to-chloride ratio^a^ was entered as continuous variable per 0.01 increase; Crude Model: didn’t adjusted for covariates; Model Ⅰ: adjusted for age, gender; Model Ⅱ: Model Ⅰ+myocardial infarction, congestive heart failure, cerebrovascular disease, chronic pulmonary disease, diabetes, renal disease, CCI, and APSⅢ; Model Ⅲ: Model Ⅱ+heart rate, MBP, RR, SPO_2_, WBC, platelet count, hemoglobin, potassium, urea nitrogen, and glucose, surgical type (on-pump vs off-pump).
